# DXA-Derived Adiposity and Lean Indices for Management of Cardiometabolic and Musculoskeletal Frailty: Data Interpretation Tricks and Reporting Tips

**DOI:** 10.3389/fresc.2021.712977

**Published:** 2021-10-20

**Authors:** Marco A. Minetto, Chiara Busso, Piera Lalli, Giulia Gamerro, Giuseppe Massazza

**Affiliations:** Division of Physical Medicine and Rehabilitation, Department of Surgical Sciences, University of Turin, Turin, Italy

**Keywords:** fat mass, frailty, lean mass, muscle mass, visceral adipose tissue

## Abstract

The proper assessment and follow-up of obesity and sarcopenia are relevant for the proper management of the complications of cardiometabolic and musculoskeletal frailty. A total body dual-energy X-ray absorptiometry (DXA) scan should be systematically incorporated in the rehabilitative routine management of patients with obesity and sarcopenia. In the former patients, the total body DXA can be used to assess the fat tissue amount and distribution, while in the latter patients, it can be used to quantify the reduction of appendicular lean mass and to investigate the inter-limb lean mass asymmetry. This tutorial article provides an overview of different DXA-derived fat and lean indices and describes a step-by-step procedure on how to produce a complete DXA report. We suggest that the systematic incorporation of these indices into routine examinations of the patients with obesity and sarcopenia can be useful for identifying the patients at risk for cardiometabolic and neuromuscular impairment-related comorbidities and for evaluating the effectiveness of pharmacological and rehabilitative interventions.

## Introduction

Frailty is a common geriatric syndrome that consists in a disorder of several inter-related systems (i.e., central nervous system, endocrine and immune systems, cardiovascular and respiratory systems, and musculoskeletal system) presenting a decrease in the physiological reserve and a failure of the homeostatic mechanisms (1, 2). The usual clinical presentation of frailty consists in a combination of the following symptoms and signs: fatigue, weight loss, frequent infections, balance and gait impairment, acute confusion, and fluctuating disability that is also known as “day-to-day instability” (2). A widely adopted operational definition of frailty includes the assessment of unintentional weight loss, muscle weakness, self-reported exhaustion, slowness, and low energy expenditure (1).

Obesity (i.e., the excessive fat accumulation that can be quantified through the increases in body mass index, waist circumference, and percentage fat mass) and sarcopenia (i.e., the age- or disease-related loss of lean/muscle mass associated with the reduction of muscle strength and performance) and their combination that is known as “sarcopenic obesity” (i.e., the co-occurrence of obesity identified by any of the above-reported definitions and of the muscle strength and lean/muscle mass reduction) (3–7) must be considered as the core pathophysiological features of cardiometabolic and musculoskeletal frailty.

The proper assessment and follow-up of obesity and sarcopenia are relevant for the management of the complications of cardiometabolic and musculoskeletal frailty: increased risk of mortality, falls and fractures, physical limitation, loss of activities of daily living (4–7).

Dual-energy X-ray absorptiometry (DXA) is widely applied in the clinical research studies and in daily clinical practice for estimating the amount and distribution of both fat and lean mass. Although this technique is not the “gold standard” method for measuring the fat and lean mass, in recent years it gained popularity as an accurate and reliable method to investigate the whole body and regional soft tissue composition (8–12).

Hologic Inc. (Bedford, MA, USA) and GE-Lunar Inc. (Madison, WI, USA) are the two dominant DXA manufacturers, which have been validated against criterion 4-compartment models. Their new high-resolution machines (Horizon densitometers for Hologic and iDXA densitometers for GE-Lunar) have recently introduced several technical advancements that will be presented in the following sections.

A total body DXA scan should be systematically incorporated in the rehabilitative routine management of patients with obesity and sarcopenia. In the former patients, total body DXA can be used to assess the fat tissue amount and distribution, while in the latter patients, it can be used to quantify the reduction of appendicular lean mass and to investigate the inter-limb lean mass asymmetry (12). A crucial point for the proper use of the DXA-derived fat and lean indices is that the physiatrists are educated about their meaning and about the importance of a correct exam acquisition and image analysis, to avoid the pitfalls and errors affecting the interpretation.

In this tutorial article, we provide a summary of the main parameters that can be obtained to assess the fat and lean mass by total body DXA. Thus, rather than providing an exhaustive overview of the approaches and techniques to investigate cardiometabolic and musculoskeletal frailty, we limit our discussion to DXA-derived variables through a description of representative cases and relative reports.

## Literature Search Strategy

This tutorial article was prepared according to the recommendations of the Scale for the Assessment of Narrative Review articles (13).

The literature research was performed to include all the relevant studies up to May 2021, by searching the Medline/PubMed database and Web of Science using the following search terms: adiposity, body composition assessment, cardiometabolic risk, DXA, fat mass distribution, frailty, lean mass asymmetry, obesity, muscle mass, visceral adipose tissue, and sarcopenia. We included the following article types: original articles, randomized controlled trials, reviews, and practice guidelines. Additional filters of the search strategy were the publication language (only articles written in English were considered), species (human studies), gender (male and female populations), and age (adult populations).

## Representative Case

**Figure 1:**
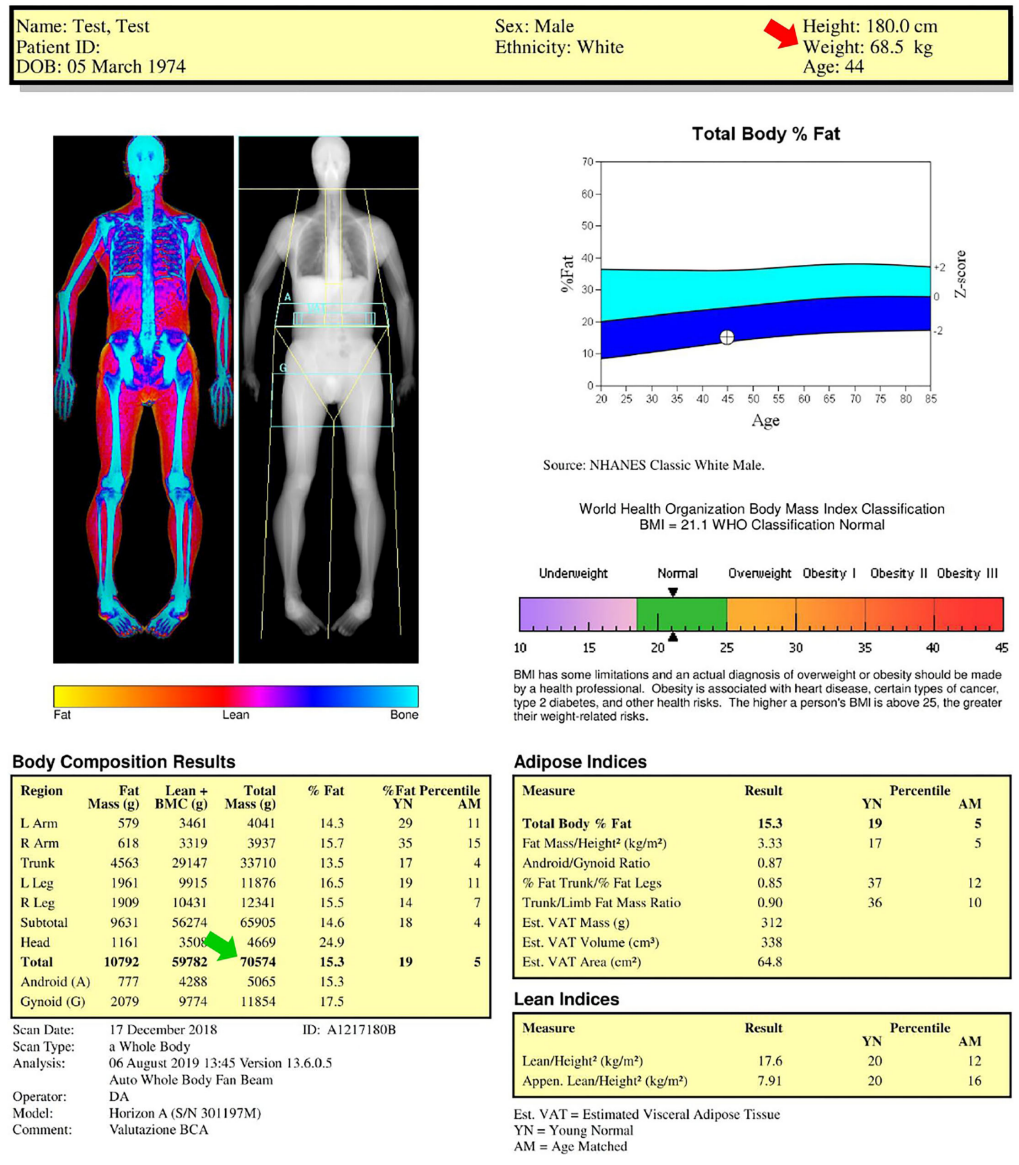
Dual-energy X-ray absorptiometry (DXA) report of body composition for one representative healthy male subject. Color image map shows the relative distribution of fat (yellow), lean tissue (orange and red), and bone (blue), while the crystal image map shows the (yellow) cut lines used to distinguish the standard regions of interest considered for body composition assessment (upper limbs, lower limbs, and trunk) and highlights the gynoid (G)/android (A) areas and the visceral adipose tissue (VAT) slice (light blue regions). Body composition results, body mass index chart, total body fat percentage chart, adipose indices, and lean indices are also reported. AM, age-matched percentile value; BMI, body mass index; BMC, bone mineral content; YN, young normal percentile value.

A representative case of total body DXA of an adult healthy male is reported in Figure 1. The color image map shows the relative distribution of bone (blue), lean tissue (orange and red), and fat tissue (yellow), while the crystal image map shows the cut lines (yellow lines) used to distinguish the standard regions of interest (upper limbs, lower limbs, and trunk) for body composition assessment and highlights the gynoid/android areas and the visceral adipose tissue slice (light blue regions). Body composition results represent the estimated amounts of the different compartments (fat mass, lean mass plus bone mineral content, and total mass). The fat mass results are also reported as percentage value (fat mass/total mass), Young Normal (YN) percentile value (also known as *T*-score), and Age-Matched (AM) percentile value (also known as *Z*-score). The *T*-score “compares” the obtained result to the database (i.e., the National Health and Nutrition Examination Survey—NHANES—DXA whole body database) of healthy young (20–29-year-old) population of the same gender, while the *Z*-score “compares” the obtained result to the same age and gender population database (14). In this representative example, the percentage total body fat was 15.3%, *T*-score was 19% (meaning that 19% of 20–29 years population database have less fat than the evaluated subject or that 81% have more fat than the evaluated subject) and *Z*-score was 5% (meaning that 5% of people of the same gender and age have less fat than the evaluated subject or that 95% of people of the same gender and age have more fat than the evaluated subject). The percentage total body fat is also represented in the total body fat percentage chart (to the right of the DXA images): this is the blue chart that plots the age of the subject and the estimated percentage fat value against the US-based NHANES dataset. The center line between the light blue and dark blue areas represents the median values (50th percentile values: in this representative example, the value of the evaluated subject is below the center line, therefore the body fat percentage is lower than the median value), while the solid area (light blue and dark blue areas) depicts ±2 SD *Z*-score of the percentage fat.

The numbers reported above the blue chart include the anthropometric data (in this representative example: height 180 cm; weight 68.5 kg, age 44 years) obtained by the technologist who collected name and birthdate, reported the gender and ethnicity, and measured the height and weight by using a scale and a stadiometer, respectively. Gender and ethnicity must be accurately verified because they may influence the *T*-score and *Z*-score values. The patient height and weight should not be asked but always carefully measured because they are used to calculate the body mass index. Moreover, the height value is used to obtain several fat and lean indices (as shown below) and the measured body weight value (red arrow in Figure 1: 68.5 kg) can be compared with the DXA-derived mass (green arrow in Figure 1: 70.5 kg) as a measure of validation.

The agreement of DXA-measured mass with scale weight is typically within 1% (15): if the agreement is above 3%, the DXA scan and weight measurement should be repeated after correction of possible analytical (e.g., thick heavy clothing must be removed, bound hair may tilt head forward, and impact chin line placement) and biological (e.g., large drinks or meals as well as strenuous exercise should be avoided before DXA scan because of their effect on the body hydration) confounders.

The image reported below the blue chart shows the classification of the weight deficiency or excess according to the following six categories of body mass index (that can be calculated as weight [kg]/height^2^ [m^2^]): (i) underweight; (ii) normality; (iii) overweight; (iv) class I (moderate) obesity; (v) class II (severe) obesity; and (vi) class III (morbid) obesity (16). The black arrowheads reported in the image indicate the value of body mass index (in this representative example: 21.1 kg/m^2^) obtained for the investigated subject from the measured values of weight and height.

It is worth mentioning that the body mass index alone cannot identify excess adiposity and establish a diagnosis of overweight or obesity in all instances. In fact, body mass index has limited inter-individual consistency for estimating the body fat percentage and distribution (17). Moreover, it may underestimate the cardiometabolic risk in some patients, such as in the elderly, while overestimating the risk in others, such as the athletes. Notably, the body mass index performs poorly in assessing the adiposity and associated health risks of athletes due to the higher muscle mass, lower body fat, and lower cardiometabolic risk at higher body mass index level (17).

The two tables under the body mass index chart list the DXA-derived adipose and lean indices, whose description is reported in the following sections.

## Adiposity Indices

The indices for the assessment of fat amount, fat distribution, and visceral adipose tissue can be obtained by DXA and may be useful for risk-stratifying patients for cardiometabolic outcomes. The fat amount assessment can be performed through the following indices: (i) percentage fat mass [%] = (fat mass [kg]/total mass [kg]) ^*^ 100; (ii) fat mass index (kg/m^2^) = fat mass (kg)/height^2^ (m^2^). It is worth mentioning that there is no consensus on the specific thresholds of either percentage fat mass or fat mass index to define obesity.

Body fat percentage cutoff points for obesity have been proposed by the WHO to be 25% for men and 35% for women (18), while the American Society of Bariatric Physicians obesity Algorithm indicated cutoff points of 25% in men and 32% in women (19).

An alternative method to define the categories of adiposity was proposed by Kelly et al. (14). They developed an obesity classification scheme by using the body mass index classification thresholds and prevalence in young adults to generate the matching classification thresholds for the fat mass index and proposed thresholds for excess fat (obesity) of 6 kg/m^2^ (9 kg/m^2^) in men and 9 kg/m^2^ (13 kg/m^2^) in women.

In addition to the above-reported measurements of adiposity, DXA report also includes the following fat distribution variables: android/gynoid fat mass ratio, trunk/limb fat mass ratio, trunk/leg fat mass ratio, and visceral adipose tissue. The relevance of these adiposity indices is related to the well-known acquisition that fat distribution in the human body may be more important for cardiometabolic health than the total fat mass (10, 18, 19). In fact, accumulations of fat in the visceral compartment and in the gluteofemoral area have opposite “metabolic” significance: “peripheral” obesity seems protective for the development of cardiovascular frailty in comparison with the “central” obesity (10, 18, 19).

The android/gynoid ratio and the android fat mass are the analog of the anthropometric measurements of the waist-to-hip ratio and waist circumference, respectively. According to the protocol of the WHO, the latter measure is taken midway between the highest point of the iliac crest and the bottom of the ribcage; cutoff points of 94 cm in men and 80 cm in women associate with the risk factors of metabolic syndrome (16). According to the protocol of the National Institutes of Health, the measure is taken at the highest point of the iliac crest; cutoff points of 102 cm in men and 88 cm in women associate with the risk factors of metabolic syndrome (20, 21). If the patient stores more fat around the android region (waist), this is a feature of the so-called “apple shape” that implies that the android/gynoid ratio is >1 in men and 0.8 in women. Conversely, if the patient stores more fat around the gynoid region (hips), this is a feature of the so-called “pear shape”.

The trunk/limb and trunk/leg fat mass ratios can be used to assess the fat redistribution (between the trunk and upper and lower limbs and between the trunk and lower limbs, respectively) in patients with lipodystrophy syndromes. In fact, about 80% of the trunk fat mass is perivisceral and about 98% of the fat mass of extremities is subcutaneous and lipodystrophy syndromes are characterized by the selective loss of subcutaneous fat, especially from the gluteal region and lower extremities, and its redistribution (i.e., accumulation of perivisceral fat resulting in marked abdominal obesity and excessive fat accumulation in the neck) (22).

According to the International Society for Clinical Densitometry official positions, DXA can be used to assess the fat distribution in the patients with HIV using antiretroviral agents associated with a risk of lipoatrophy (23). Different cutoffs of the trunk/leg fat mass ratio have been proposed to diagnose lypodistrophy in the patients with HIV of different ethnic groups. In a French study performed in male patients, Bonnet et al. (24) proposed the cutoff of 1.5, while Beraldo et al. (25) proposed a lower cutoff (1.26) in male Brazilian patients. Freitas et al. (26) identified in a Portuguese population of patients of both genders the following gender-specific cutoffs: 1.961 in men and 1.329 in women.

In the past few years, both GE and Hologic developed new software packages (CoreScan for GE and InnerCore for Hologic) for the assessment of the visceral adipose tissue, that is obtained by subtracting the subcutaneous fat (located on both side of the abdominal cavity) of the android area from the android fat mass. The Hologic's software automatically locates the outer and inner margins of the abdominal wall in a 5 cm region of the abdomen (the lower border of this region coincides with the L4 vertebral level). The software measures the total abdominal fat and subcutaneous fat (from both sides) of the region of interest and reports the visceral fat content as the difference between these measurements. The region of interest considered by the GE CoreScan software to estimate the visceral adipose tissue includes the whole android area (defined by the pelvic line as lower boundary, trunk lines as lateral boundaries, and a horizontal line as the upper boundary identified by measuring the 20% of the distance between the pelvis line and head line). Although different estimates of visceral adipose tissue (i.e., mass, volume, and area) can be obtained by DXA, several studies suggested that the visceral adipose tissue area can be used to distinguish between the following two classes of cardiometabolic frailty risk: (i) 100–159 cm^2^: increased risk, (ii) ≥160 cm^2^: high risk (10, 27, 28).

## Lean Indices

Total body lean mass and appendicular lean mass (ALM: the lean mass of the upper and lower limbs) are the main parameters obtained through DXA to assess lean mass. Their absolute values can be normalized to the height^2^ (or to the body mass index) to account for allometric differences in body size, thus obtaining the lean mass index (LMI) or the appendicular lean mass index (ALMI) that enable the comparisons among the different subjects independently of their body size.

Different cutoff points have been proposed (29–32) to discriminate between the normal and low lean mass (Table 1). Given that the cutoff thresholds derived by Suetta et al. (32) differed from earlier reference data (29–31), the authors underlined the importance of obtaining updated and local reference materials (32). We recommend the use of the cutoffs proposed in the last revision of the European Working Group of Sarcopenia in Older People (EWGSOP-2) consensus (29) that have been produced for different parameters (ALM and ALMI: as shown in Table 1) as rounded figures to facilitate their use in the clinical practice and to increase harmonization of sarcopenia studies.

**Table 1:** Cutoff points proposed to discriminate between the normal and low lean mass.

**Variable**	**Men**	**Women**	**Reference**
ALMI [kg/m^2^]	7.26	5.45	(29)
ALM [kg]	19.75	15.02	(30)
ALM / BMI	0.789	0.512	(30)
ALM [kg]	20	15	(31)
ALMI [kg/m^2^]	7.0	5.5	(31)
LMI [kg/m^2^]	14.58	12.14	(32)
ALMI [kg/m^2^]	6.60	5.03	(32)

*ALM, appendicular lean mass; ALMI, appendicular lean mass index; BMI, body mass index; LMI, lean mass index*.

Dual-energy X-ray absorptiometry can also be useful in studying the lean mass asymmetries between the two sides of both the upper and lower limbs. Although the indices of lean mass asymmetry are usually not included in the DXA reports, they can easily be calculated and included in the final report. For example, the limb asymmetry index can quickly be obtained according to the following formula (33):


$$\begin{array}{l} {}[Difference\;in\;lean\;mass\;between\;the\;two\;sides/0.5\;*\;\\ (sum\;of\;the\;lean\;mass\;of\;the\;two\;sides)]\;*\;100 \end{array}$$

The upper and lower limb asymmetry indices of the representative case reported in Figure 1 are 4.3 and 5.4%, respectively.

Different equations can be used for calculating the interlimb asymmetry of the lean mass: it is worth mentioning that no consensus exists on the reference index and that normative data for the lean mass asymmetry index of different populations are not available.

## Calibration Issues

The body composition results are calibration dependent and the results provided by different instruments can vary. In the 1999–2004 NHANES, the DXA scans were performed and analyzed using Hologic QDR4500 A fan-beam densitometer. In a very relevant manuscript, Schoeller et al. (34) combined different studies comparing the NHANES-derived DXA data and criterion methods for body composition assessment (i.e., underwater weighing, total body water assessment by deuterium dilution, and four compartment models) and found in a large (*n* = 1,195) group of patients that DXA overestimated the lean mass and underestimated the fat mass compared with the criterion methods. On this basis, Schoeller et al. (34) suggested that the DXA (QDR4500 A)-derived lean soft tissue mass estimates collected in the NHANES survey must be recalibrated through the application of a “correction factor”: this calibration adjustment, known as the “NHANES BCA calibration,” consists in the reduction of the DXA-derived lean mass by a factor of 0.946. This NHANES calibration was applied before the results were publicly released: the adjusted percentage of fat values, about 3–5% above that measured, have since been used to set normative adiposity range (14).

**Figure 2:**
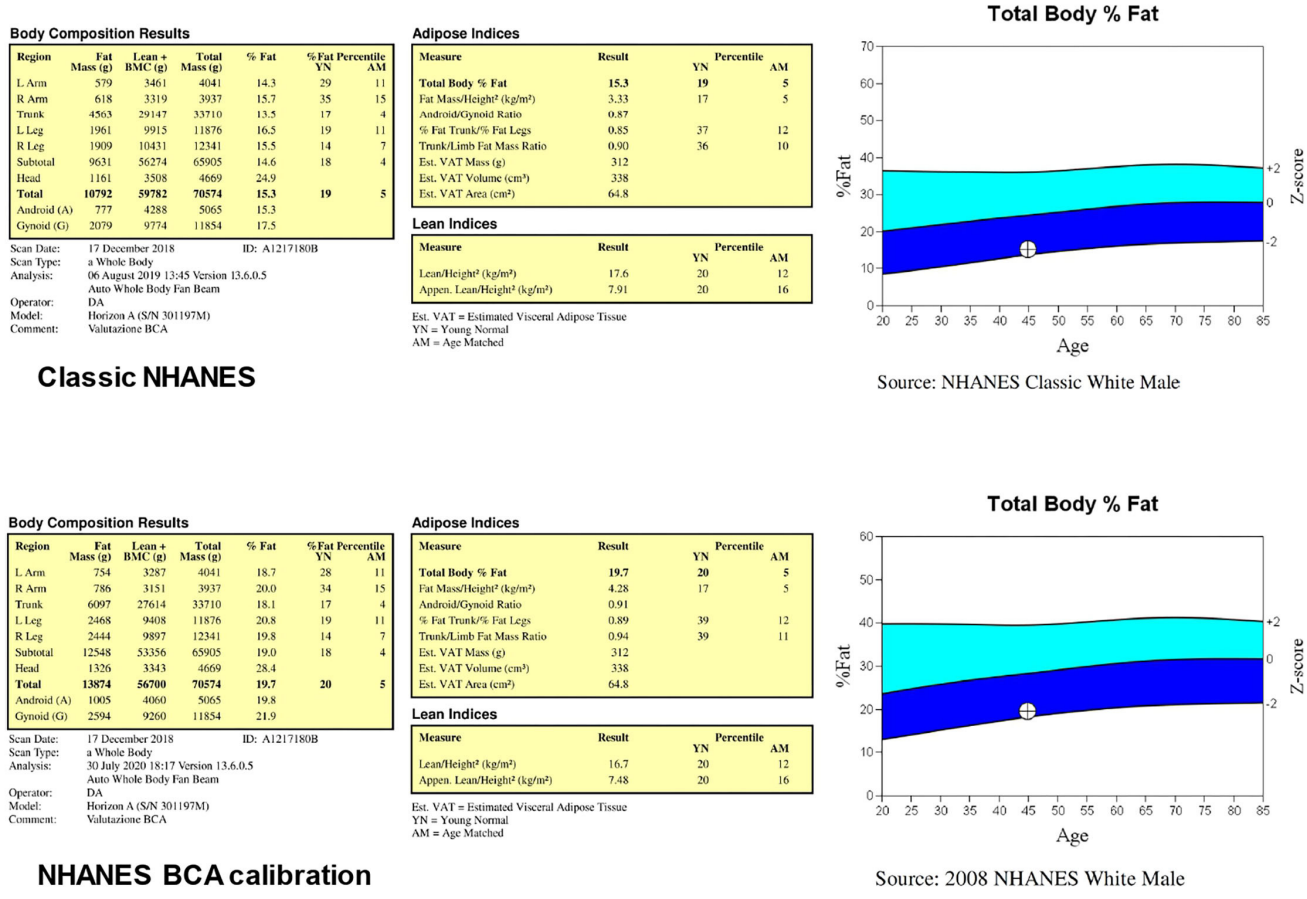
Results of the body composition analyses performed (for the same subject of Figure 1) through the two body composition calibration methods called “classic NHANES” and “NHANES BCA calibration”.

The use of Hologic devices allows to perform the analysis of total body scans on either two body composition calibration methods called “classic NHANES” and “NHANES BCA calibration.” Figure 2 reports the results of the body composition analyses performed with the two calibration methods for the same representative subject of Figure 1: total body percentage fat increases from 15.3% with the classic NHANES to 19.7% with the NHANES BCA calibration, fat mass index increases from 3.33 kg/m^2^ with the classic NHANES to 4.28 kg/m^2^ with the NHANES BCA calibration, while ALMI decreases from 7.91 kg/m^2^ with the classic NHANHES to 7.48 kg/m^2^ with the NHANES BCA calibration.

A recent study by Ng et al. (35) compared, in a small (*n* = 23) group of healthy adults, DXA-derived percentage fat mass and fat mass estimates obtained with a four-component body composition assessment: they found that enabling the NHANES BCA calibration resulted in overestimated DXA fat values compared with the criterion method. Although the authors properly acknowledged that the small sample size limited the strength of their study, they suggested that re-evaluation of DXA body composition calibration standard is required to optimize the accuracy of the method. Consistently, the recent studies performed with a Hologic device reported that the NHANES BCA calibration option was disabled for scan analysis (36–39). Although further studies are required to establish whether the NHANES BCA calibration option should always be disabled or must be used in the selected subgroups of patients (e.g., obese patients), it is recommended that a serial evaluation of the same patient to assess the body composition changes is performed with the same software configuration (that should be specified in the report).

## Representative Reports and Conclusion

This tutorial article provided an overview of the different DXA-derived fat and lean indices and described a step-by-step procedure on how to produce a complete DXA report.

According to the International Society for Clinical Densitometry (40), DXA reports should include the manufacturer, model, a statement regarding the scan factors that may adversely affect the acquisition/analysis quality, a diagnostic interpretation of the DXA measurements (e.g., “overweight” in case of body mass index values in the range 25.0–29.9 kg/m^2^) and, in the case of follow-up DXA report, statement regarding which previous or baseline study is being used for comparison. Moreover, the optional data (e.g., waist circumference) acquired by the technologist before/after DXA scan can also be included in the report because of their possible relevance for the research purposes (41–43).

**Figure 3:**
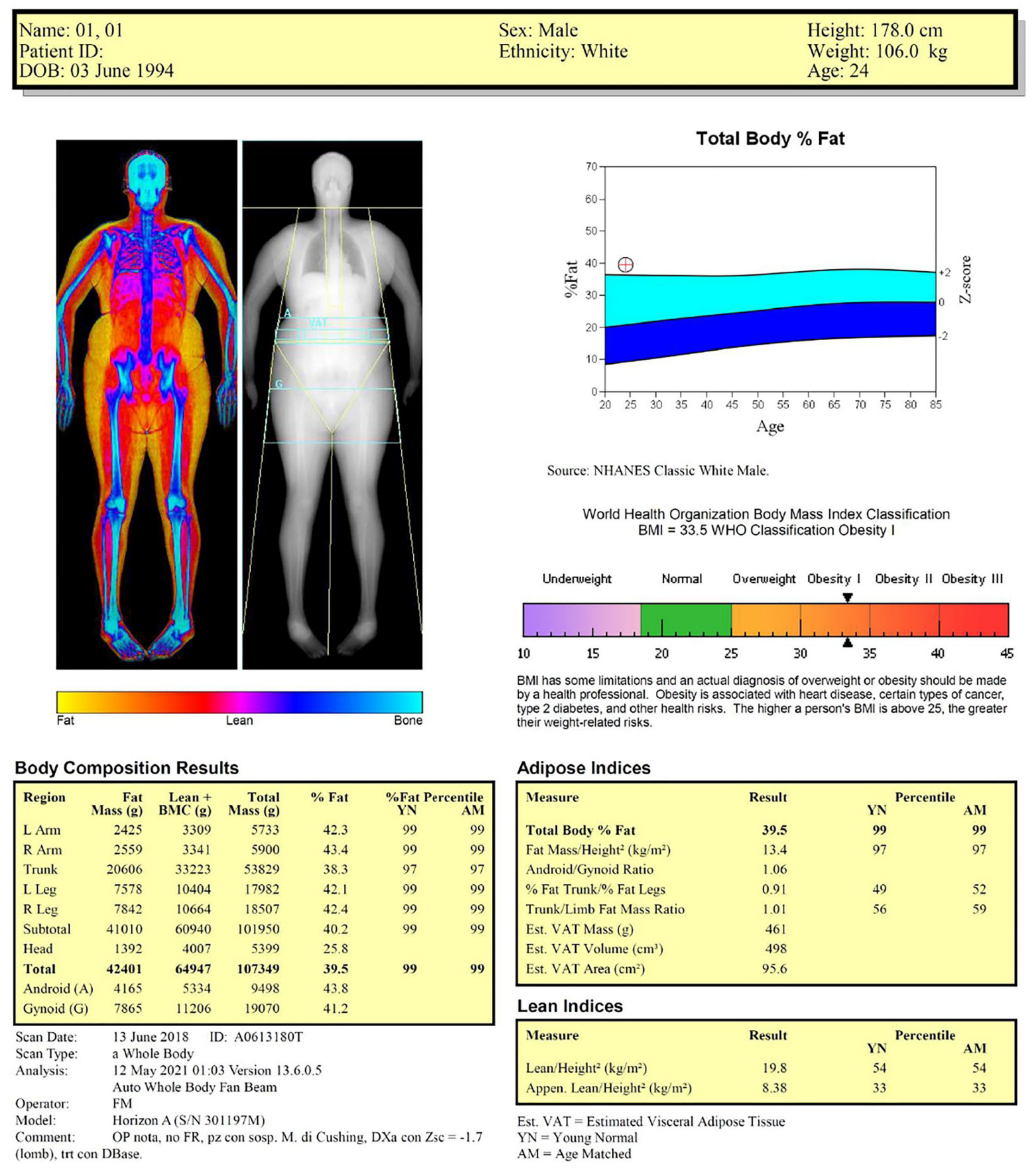
Dual-energy X-ray absorptiometry report of body composition for one representative obese male subject.

Table 2 shows the examples of reports for the two representative cases shown in Figure 1 (healthy lean male subject) and Figure 3 (obese male subject): the two reports include the previously described anthropometric variables and DXA-derived fat and lean indices. We suggest that the systematic incorporation of these variables and indices into routine examinations of the patients with obesity and sarcopenia can be useful for identifying the patients at risk for cardiometabolic and neuromuscular impairment-related comorbidities and for evaluating the effectiveness of pharmacological and rehabilitative interventions.

**Table 2:** Examples of reports for the two representative cases of Figure 1 (healthy male) and Figure 3 (obese male).

**Subject 1 (Representative Case of** **Figure 1****)**	
Device	Horizon A
Scan type	Total body
Scan analysis	NHANES BCA calibration option disabled
Measurement validation (i.e., agreement of DXA-measured mass with scale weight)	2.9%
Anthropometry	• Normal body mass index (21.1 kg/m^2^) • *Body surface area: 1.87 m^2^* • *Normal waist circumference (83 cm)* • *A body shape index (ABSI): 0.081: ABSI Z-score:−0.141, relative risk: 0.90 (waist circumference-related mortality risk: 10% lower than the average population of 44 year old males)*
Adipose indices	• Normal percent fat mass • Normal fat mass index • Gynoid fat mass distribution • Normal visceral adipose tissue with low cardiometabolic frailty risk
Lean indices	• Normal appendicular lean mass (25.6 kg) • *Total body skeletal muscle mass: 28.7 kg* • Symmetric lean mass distribution for the upper limbs (asymmetry index: 4.3%) • Symmetric lean mass distribution for the lower limbs (asymmetry index: 5.4%)
**Subject 2 (Representative Case of** **Figure 3****)**	
Device	Horizon A
Scan type	Total body
Scan analysis	NHANES BCA calibration option disabled
Measurement validation (i.e., agreement of DXA-measured mass with scale weight)	1.2%
Anthropometry	• Body mass index = 33.5 kg/m^2^ (obesity class I) • *Body surface area: 2.23 m^2^* • *Increased waist circumference (106 cm)* • *A body shape index (ABSI): 0.076: ABSI Z-score:−0.482, relative risk: 0.88 (waist circumference-related mortality risk: 17% lower than the average population of 24 year old males)*
Adipose indices	• Increased percent fat mass • Increased fat mass index (obesity class II) • Android fat mass distribution • Normal visceral adipose tissue with low cardiometabolic frailty risk
Lean indices	• Normal appendicular lean mass (26.5 kg) • *Total body skeletal muscle mass: 30.4 kg* • Symmetric lean mass distribution for the upper limbs (asymmetry index: 0.8%) • Symmetric lean mass distribution for the lower limbs (asymmetry index: 2.4%)

*Optional data are reported in italics: these data can be acquired by the technologist before/after DXA scan (e.g., waist circumference) and can by calculated by the physician who prepares the report through previously validated equations:*

(i) Du Bois and Du Bois (41):

body surface area [m^2^] = 0.007184 × weight [kg] ^0.425^ × height [cm] ^0.725^.

(ii) Krakauer et al. (42):

A body shape index (ABSI) = WC [m]/(BMI [kg/m^2^] ^2/3^ × height [m] ^1/2^).

(iii) Kim et al. (43):

Total body skeletal muscle mass [kg]: 1.18 × ALM [kg] − (0.03 × age [years]) − 0.14.

ALM, appendicular lean mass; BMI, body mass index; WC, waist circumference.

## Author Contributions

MM and GM: conceptualization. CB and PL: data acquisition (literature search and study selection), analysis, and interpretation of data (literature). MM and GG: drafting of the manuscript. MM, CB, and PL: writing—review and editing the manuscript. All authors contributed to the article and approved the submitted version.

## Funding

The authors' work related to this review was supported by the grants from the University of Turin (Research Fund ex-60%), the Fondazione CRT (Turin, Italy), and by the Ministry of Education, University and Research (MIUR) under the program Dipartimenti di Eccellenza ex L. 232/2016 to the Department of Surgical Sciences, University of Turin, Italy.

## Conflict of Interest

The authors declare that the research was conducted in the absence of any commercial or financial relationships that could be construed as a potential conflict of interest.

## Publisher's Note

All claims expressed in this article are solely those of the authors and do not necessarily represent those of their affiliated organizations, or those of the publisher, the editors and the reviewers. Any product that may be evaluated in this article, or claim that may be made by its manufacturer, is not guaranteed or endorsed by the publisher.
